# Compliance with infection prevention and control practices and associated factors among healthcare workers in Tanzania: Experience from a secondary level-referral hospital

**DOI:** 10.1371/journal.pone.0337254

**Published:** 2026-06-11

**Authors:** Cesilia Charles, Lutengano Mkonongo, David Masanja, Pendo Edward, Damian Maruba, Philipo Felix Mwita, Baraka Nkondo, Frank Elisha, Edward Bucheye, Abel Nyika, Avent Kalumiana, Emmanuel Amsi, Elly Ambikile, Nathanael Sirili, Joshua Mollel, Bernard Njau, Radenta Bahegwa, Deogratias Banuba

**Affiliations:** 1 Research and Publication Unit, Katavi Regional Referral Hospital, Katavi, United Republic of Tanzania; 2 Health Quality Improvement Unit, Katavi Regional Referral Hospital, Katavi, United Republic of Tanzania; 3 Obstetrics and Gynaecology Department, Katavi Regional Referral Hospital, Katavi, United Republic of Tanzania; 4 Surgical Department, Katavi Regional Referral Hospital, Katavi, United Republic of Tanzania; 5 Internal Medicine Department, Katavi Regional Referral Hospital, Katavi, United Republic of Tanzania; 6 Emergency Department, Katavi Regional Referral Hospital, Katavi, United Republic of Tanzania; 7 Paediatric Department, Katavi Regional Referral Hospital, Katavi, United Republic of Tanzania; 8 Laboratory department, Katavi Regional Referral Hospital, Katavi, United Republic of Tanzania; 9 Department of Development Studies, University of Health and Allied Sciences, Dar Es Salaam, United Republic of Tanzania; 10 School of Public Health, KCMC University, Kilimanjaro, United Republic of Tanzania; 11 Health Quality Assurance Unit, Ministry of Health, Dodoma, United Republic of Tanzania; World Health Organization, CONGO

## Abstract

**Introduction:**

Compliance with Infection Prevention and Control practices remains a key challenge, affecting the safety of both patients and healthcare workers. Poor compliance raises the risk of Hospital-Acquired Infections (HAIs) and antimicrobial resistance (AMR).

**Objective:**

This study aimed to assess compliance levels and factors associated with infection prevention and control practices among HCWs at Katavi Referral Regional Hospital (KRRH) in Tanzania.

**Methods:**

A hospital-based cross-sectional study was conducted among 195 healthcare workers from July 24 to August 23, 2025. Questionnaires and observation checklists were used to collect sociodemographic data, compliance levels, individual-level factors, hospital-level factors, and the availability of IPC supplies. A validated Compliance with Standard Precautions Scale (CSPS) tool, developed by the WHO, was used to measure compliance levels. Data were analysed in STATA (version 15.0), using bivariate and multivariate modified Poisson regression models. Adjusted Prevalence Ratio (APR) with 95% Confidence Interval (CI) was used to assess factors associated with IPC compliance.

**Results:**

The study revealed that the overall compliance with IPC practices among healthcare workers was 68.9%. Only 39.0% of HCWs demonstrated high overall compliance with IPC practices (<80%), while 61% had low overall compliance (>80%). Also, factors significantly associated with compliance with IPC practices were doctor profession (APR: 0.32;95% CI:0.19,0.57), blood/body fluid exposure (APR: 1.55;95% CI:1.095,2.19), motivation at workplace (APR: 1.43;95% CI:1.02,2.02), supportive supervision (APR: 1.92;95% CI:1.09,3.38), and presence of IPC committee (APR: 1.61;95% CI:1.07,2.40). The most common available IPC supplies were hand hygiene items, personal protective equipment, and waste management items (100%). However, some IPC supplies were unavailable, including water (44.4%) and soap (55.6%) in latrines.

**Conclusion:**

Overall compliance with IPC practices among HCWs remained suboptimal. Improving compliance requires strengthening IPC governance through functional IPC committees, enhancing supportive supervision and motivation, and addressing persistent infrastructural and resource gaps within health facilities.

## Introduction

Hospital-acquired infection (HAI) is the most common adverse event in healthcare delivery systems globally, threatening the health of patients and healthcare workers. In 2024, the WHO global report highlighted that HAIs kill seven patients per 100 in high-income countries and 15 patients per 100 in lower- and middle-income countries (LMICs) annually [1]. In Sub-Saharan African countries, the HAI rate is 12.9%, of which the highest burden (19.7%) has been observed in East Africa [2–6]. In Tanzania, the rate of HAI is 14.8% higher than in the developed world [7]. The main cause for the high HAI rate is non-compliance with IPC practices among HCWs [8]. Additionally, the lack of an IPC committee, IPC training, and IPC policies/guidelines impedes their compliance [9–11].

According to the World Health Organization (WHO), quality healthcare is regarded as patient-centred, safe, effective, timely, efficient, and equitable [12]. Within this framework, infection prevention and control (IPC) is recognized as a fundamental element in delivering high-quality care by addressing HAIs, antimicrobial resistance (AMR), and emerging pathogen containment [13–15]. IPC practices are designed to protect patients, staff, and visitors from Hospital Acquired Infections (HAIs) and exposure to disease-causing agents (germs) that may be present in the healthcare environment [16]. These practices include hand hygiene, PPE use, handling of sharp devices, immunization, post-exposure prophylaxis, and isolation enacted for the prevention of HAIs [1].

Nearly 70% of all HAIs can be prevented using available evidence-based IPC strategies [17]. Regrettably, only 34% of WHO member states in 2021 had a fully implemented IPC programme across their country, and among them, only 19% reported having a system in place to monitor the effectiveness and compliance with the implemented prevention and control activities [6]. Low IPC compliance is associated with increased hospital-acquired infections, antimicrobial resistance, and even death [1]. Beyond these adverse effects, low compliance with IPC poses a significant risk for mental health disorders, such as anxiety, depression, adjustment disorder, panic attacks, post-traumatic stress disorder, and economic burden to health systems and families as a result of HAI and AMR [18].

Despite several interventions carried out in Tanzania that were aimed at improving compliance with IPC practices, including disseminating a new version of the national IPC guideline, which was published in 2018, compliance with IPC practices remains far from satisfactory in most hospital settings, suggesting that other factors could be responsible for low compliance with IPC practices [19]. Therefore, this study was designed to assess compliance with IPC practices and associated factors among healthcare workers at Katavi Regional Referral Hospital in Tanzania. These findings are expected to increase the knowledge among healthcare workers on the magnitude and factors influencing compliance with IPC practices and enable them to plan and implement rational interventions. Moreover, the findings may highlight knowledge gaps to be filled in future studies.

## Materials and methods

### Study design

A facility-based cross-sectional study was conducted among healthcare workers (HCWs) using a quantitative approach to assess the compliance levels with Infection Prevention and Control (IPC) and their associated factors.

### Study setting and period

Data were collected from July 24 to August 23, 2025, at KRRH, one of 28 Tanzanian government regional referral hospitals located in Mpanda Municipality. According to the 2022 Census, the hospital serves a catchment population of 1,152,958 (26). It offers a range of inpatient and outpatient services, including medical, surgical, obstetrics, gynaecology, child health, laboratory, and rehabilitation services. It receives patients from Katavi’s councils, neighbouring regions, and the Democratic Republic of Congo. KRRH has 445 employees of various cadres, which is below the minimum range of 481, a recommended staffing requirement for a Regional Referral Hospital according to Tanzania’s MoH guidelines [20]. Out of 445 employees, 366 (82.2%) are doctors, nurses, and laboratory practitioners, and the rest, 79 (17.8%) are supportive staff (pharmacists, environmental officers, human resource officers, finance officers, etc.).

### Study population

The study enrolled healthcare workers who were permanently employed at KRRH for a minimum of six months and were involved in patient care. Volunteers, medical students, and HCWs who were on leave or unavailable during the data collection period were excluded.

### Sample size determination

The sample size was determined using the single population formula by taking a prevalence value of 54% (0.54) from a study conducted at St Francis Regional Referral Hospital in Tanzania, by considering a confidence interval (CI) of 95%, a margin of error of 5% and using 10% for non-response rate practices [21]. N was calculated by using the Cochran formula, d, and adding 10% non-respondents.


$$\begin{array}{c} \frac{{\text{1}.\text{9}\text{6}}^{\text{2}\;}\times \text{0}.\text{5}\text{4}\times (\text{1}-\text{0}.\text{5}\text{4})}{{\left(\text{0}.\text{0}\text{5}\right)}^{\text{2}}}=\text{3}\text{8}\text{1}+(\text{3}\text{8}\text{1}\times \text{1}\text{0}\%)(\text{non}-\text{response rate})=\text{4}\text{1}\text{9}, \end{array}$$

Where n = sample size; P = proportion Zα/2=at 95% CI, which is equal to 1.96

d = margin of error or desired precision, which is equal to 5% = 0.05, after that 10% non-response rate; total sample size was 419

Since the HCWs population (N = 366) was known from the human resource registry and was smaller than the minimum sample size calculated from the Cochrane formula, the sample size formula for a finite population was used to adjust the sample population.

n = N*X / (X + N – 1), where:

N =the population size (366)

X = the minimum sample size calculated by the Cochrane formula

Thus:

n = 366*419/ (419 + 366-1) = 195

The adjusted sample size was 195 HCWs (Table 1), with a 100% response rate.

**Table 1:** Adjusted sample size for equal representation of participants.

Professions	Population per cadre	Percentage (%)	Proportional sample size
Doctors	83	22.7	83/366 x195 = 44
Nurses	252	68.8	252/366 x195 = 134
Laboratory practitioners	31	8.5	31/366 x195 = 16
**Total**	**366**	**100**	**195**

### Sampling procedures

A stratified random sampling method was used to select study participants based on their profession. Each subgroup was chosen with an equal and independent chance for all HCWs in the sample by employing a simple random sampling technique. The respondents were selected using a computer-generated random number.

### Study variables

**Independent variables were:** social demographic characteristics, working experiences, years of working experience, history of needle stick injury, hepatitis B vaccination, supportive supervision, IPC training, IPC motivation at the working workplace, availability of IPC supplies, and presence of an IPC committee.

**The dependent variable was compliance with standard IPC practices,** defined as the extent to which HCWs follow IPC practices designed by WHO to ensure patient safety, infection control, and ethical practices within the healthcare setting.

### Measurement of the dependent variable

The dependent variable was the overall compliance with IPC practices, which was dichotomous, categorized as either high overall compliance or low overall compliance. A four-point Likert scale (Never, Often, Sometimes, and Always) was used to measure the 20 items for IPC practices, including hand washing, waste management, decontamination, and the use of PPE. Items 2, 4, 6, and 15 were negatively stated, while the rest of the items were positively stated. A score of 1 was given to an “always” response in positively worded statements and “never” option in negatively worded statements, while 0 was given for the rest of the responses, giving a total possible range score of 0–20. The internal reliability score was Cronbach’s alpha = 0.8, indicating acceptable internal reliability. The compliance was quantified as the mean percentage value derived from the 20 items. The dichotomous dependent variable was categorized as high overall compliance ≥80% (≥16/20) and low overall compliance<80% (<16/20). This categorization was based on Tanzania’s national guidelines for the recognition of the implementation status of quality improvement initiatives in health facilities, including IPC improvement initiatives [22].

### Data collection

A validated and pre-tested self-administered questionnaire adapted from the WHO IPC assessment framework [23], was used to collect sociodemographic characteristics, compliance with IPC practices, and factors that could potentially be associated with compliance among HCWs at KRRH. Using Kobo Toolbox, twelve trained HCWs collected data, and two supervisors were employed to follow up on the data collection processes. Additionally, an observational checklist was developed to gather information on the availability of IPC supplies in nine departments where HCWs were working.

### Data analysis

Data were collected using Kobo Toolbox exported in excel format (S1 Data) and analysed using STATA version 15.0. Categorical variables were summarized as frequencies and percentages, while continuous variables were summarized as means and standard deviations (SD). The chi-square test was performed to determine the association between independent variables and the dependent variable at a significance level of P-value ≤ 0.05. Modified Poisson regression with robust standard errors was used to estimate the prevalence ratio (PR) and adjusted prevalence ratios (APR) with 95% confidence intervals (CI), because the binary outcome had a prevalence greater than 10% for which logistic regression may overestimate effect sizes. Variables with p-value <0.2 in the bivariate analyses and those reported in previous literature were included in the multivariable model to control for potential confounders. Statistical significance was set at p < 0.05.

### Ethical consideration

The ethical clearance was obtained from the Institutional Review Board (IRB) of the Medical Research and Ethics Committee (MMREC) in Mbeya, Tanzania (SZEC-2439/ R.A/25/11). Permission to conduct the study was also secured from the Research and Publication department of Katavi Regional Referral Hospital (KRRH). Study participants provided written informed consent to participate voluntarily. The authors declared that the information presented in this research article is original work that has not been presented elsewhere

## Results

### Demographic characteristics of healthcare workers

Of 195 HCWs included in the study, the majority were nurses, 69.2% (135/195), while the least were laboratory practitioners, 8.7% (17/195). The mean age of respondents was 31.6 (±5.5) years, with a male preponderance of 57.9% (113/195). Most HCWs, 47.7% (93/195), had a diploma-level education, and 44.1% (86/195) had worked between 2 and 5 years in healthcare service delivery. (Table 2) shows the characteristics of the study participants.

**Table 2:** Characteristics of the study participants (N = 195).

Variable	Frequency (n)	Percentage (%)
**Age (Years)**		
18–30	97	49.7
31–40	84	43.1
41–50	14	7.2
Mean (SD)	31.6 (±5.5)	
**Sex**		
Female	82	42.1
Male	113	57.9
**Professions**		
Doctor/Clinician	43	22.1
Nurse (RN/ANO/Medical Attendant)	135	69.2
Laboratory Technician	17	8.7
**Education level**		
Bachelor’s degree	73	37.4
Diploma	93	47.7
Certificate	29	14.9
**Working Experience (Years)**		
6months to 1 year	55	28.2
2 to 5 years	86	44.1
6 years and above	54	27.7

### Compliance with the infection prevention and control standards precautions

**Fig 1:**
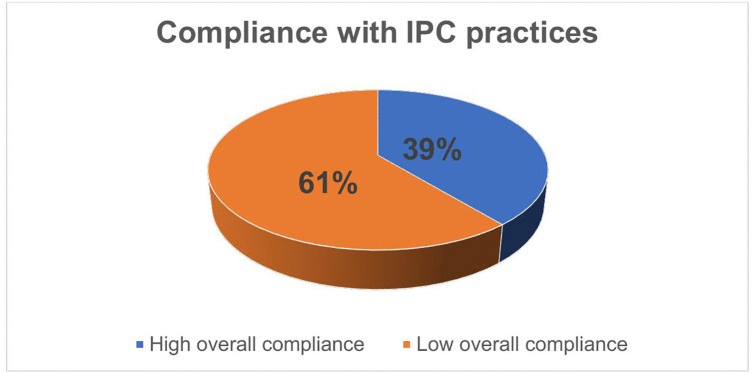
Overall infection Prevention and Control (IPC) compliance among healthcare workers. Percentage of study participants (*N* = 195) categorized by overall compliance levels. High overall compliance is defined as scoring ≥80% (≥16/20) and low overall compliance <80% (<16/20), in accordance with the United Republic of Tanzania national guidelines for quality improvement initiatives in health facilities.

The overall mean compliance with IPC among HCWs was 68.9%. Out of 195 study participants, 39% (76/195) reported a high overall compliance with IPC practices, and 61% (119/195) had a low overall compliance, as illustrated in (Fig 1).

### Compliance with IPC standard precautions

The majority of HCWs, 88.2% (172/195), reported the highest compliance with wearing gloves when exposed to body fluids, blood products, and patient excretion; however, more than half, 53.8% (105/195), of HCWs do not take a shower in case of extensive splashing, even after they have put on PPE. Nearly 68.0% (132/195) of HCWs use water alone for handwashing, and only 35.9% (70/195) reported that it is incorrect for the sharps box to only be disposed of when it is full. The compliance with IPC precautions among HCWs is detailed in the comprehensive monitoring sheet (S1 Table).

### Factors associated with compliance with IPC practices among healthcare workers

In multivariable modified Poisson regression analysis, five factors were significantly associated with compliance with IPC practices among HCWs at KRRH. Doctors and clinicians were less likely to comply with IPC practices compared to nurses (APR: 0.32;95% CI:0.19,0.57, p < 0.001). HCWs who reported exposure to body fluids were more likely to comply with IPC practices than those without such exposure (APR: 1.55;95% CI:1.095,2.19, p = 0.013). IPC motivation at the workplace was positively associated with IPC compliance, with motivated HCWs showing higher compliance compared with those who were not motivated (APR: 1.43;95% CI:1.02,2.02, p = 0.040). Additionally, HCWs who received annual IPC supportive supervision were more likely to comply with IPC practices than those who received no supervision (APR: 1.92;95% CI:1.09,3.38, p = 0.023). Similarly, the presence of an IPC committee was significantly associated with higher compliance (APR: 1.61;95% CI:1.07,2.40, p = 0.021), as illustrated in (Table 3).

**Table 3:** Factors associated with IPC compliance among HCWs (N = 195).

Variable	Compliance		P-value (X^2^ test)	APR (95% CI)	P value
	Low Compliance n (%)	High Compliance n (%)			
**Age (years)**					
18-30	63(65)	34(35)	1.599	–	
31-40	48(58.3)	35(41.7)			
41-50	7(50.0)	7(50.0)			
**Professions**					
Nurse	72(53.3)	63(46.7)	**0.002**	1	
Doctor/Clinician	36(83.7)	7(16.3)		0.32(0.19 −0.57)	**<0.001**
Laboratory Technician	11(64.7)	6(35.3)		0.68(0.34 −1.39)	0.300
**Sex**					
Female	51(62.2)	31(37.8)	0.081	–	
Male	68(60.2)	45(39.8)			
**Needlestick Injury**					
**No**	**61(69.3)**	**27(30.7)**		**1**	
Yes	55(51.4)	52(48.6)	**0.011**	1.26(0.90- 1.76)	0.176
**Exposed to blood/body fluids**					
No	60(72.3)	23(27.7)		1	
Yes	56(50.0)	56(50.0)	**0.002**	1.55(1.10-2.19)	**0.013**
**Hepatitis B Vaccination**					
No	36(45.6)	43(54.4)		1	
Yes	80(69.0)	44(31.0)	**0.001**	0.81(0.59- 1.11)	0.185
**IPC trainings**					
No training	77(66.4)	39(33.6)	**0.018**	1	
Received Training	39(49.4)	40(50.6)		1.27(0.95- 1.72)	0.112
**Motivation at the Workplace**					
No	64(72.3)	24(27.3)	**0.001**	1	
Yes	52(48.6)	55(51.4)		1.43(1.02- 2.02)	**0.040**
**Frequency of IPC supportive supervision**					
No supervision	41(80.4)	10(19.6)		1	
Monthly	25(51.0)	24(49.0)		1.40(0.76 −2.57)	0.284
Quarterly	21(72.4)	8(27.6))	**<0.001**	0.89(0.42 −1.86)	0.754
Annually	29(43.9)	37(56.1)		1.92(1.09 −3.38)	**0.023**
**Presence of the IPC committee**					
No	54(76.1)	17(23.9)		1	
Yes	62(50.0)	62(50.0)	**<0.001**	1.61(1.07- 2.40)	**0.021**

### Availability of IPC supplies at KRRH departments

The availability of IPC supplies plays a crucial role in how HCWs implement IPC practices in a health facility. During the observation of IPC supplies at KRRH, nine departments (Emergency, neonatal, Intensive care unit, paediatric, medical, surgical, theatre, laboratory, and maternity) were assessed for the availability of IPC supplies using an observational checklist. These departments were selected because they represent areas where IPC practices are most critical for preventing HAI.

Based on the observation (Table 4), the most available IPC supplies in the department were hand hygiene items (100%), injection safety (100%), sterilization (100%), and waste management (100%). The majority of departments (7) had PPE supplies like face masks, disposable gloves, and gowns/aprons/lab coats (100%), while two departments were missing protective eyewear. Nearly all departments had functional and clean latrines, while five departments had no soap, and four departments lacked constantly running water for hand washing in the latrine. Overall, the availability of essential IPC supplies in most departments supports the observed levels of IPC compliance among HCWs, while identified gaps, particularly in protective eyewear, soap, and running water, may have constrained full compliance in specific IPC components.

**Table 4:** Availability of IPC supplies at KRRH departments via an observational checklist.

Component	Item	Availability of an item N (%)	
		Yes	No
**Hand hygiene**	Alcohol hand rub, Soap at washing points, Detergent, Water, Hand washing vessel	**9(100)**	
**Personal Protective Equipment:**	Protective eyewear	**7(77.8)**	**2(22.2)**
	Face mask, Disposal gloves, Gown/Apron/lab coat	**9(100)**	
**Injection safety**	Ordinary single-use syringes, Sharp disposal containers/safety boxes	**9(100)**	
**Sterilization**	Functional Autoclaves (with pressure gauge working)	**9(100)**	
**Waste Management**	Color-coded bins, Waste disposal containers, Strong gloves for waste disposal	**9(100)**	
	Waste pit for non-infectious waste and sharps	**9(100))**	
**Latrine**	Functional and clean	**8(88.9)**	**1(11.1)**
	Soap	**4(44.4)**	**5(55.6)**
	Water is available for hand washing	**5(55.6)**	**4(44.4)**

## Discussion

This study aimed to assess overall compliance with Infection Prevention and Control Practices (IPC) and their associated factors among HCWs at KRRH.

The low overall compliance results observed in this study align with related studies conducted in Nigeria (59%), Rwanda (64.5%), Lesotho (63.6%), Ethiopia (65%), and parts of Tanzania, such as Songwe region (66%) and St. Francis Regional Referral Hospital (54%) [21,24–26]. This may be attributed to ongoing challenges, such as inadequate PPE supplies, a lack of motivation, and negative attitudes toward IPC practices, which are prevailing in many health facilities in lower- and middle-income countries, including Tanzania. However, our study results are higher than those in similar studies conducted in Iran (34%) and Bangladesh (36%), but lower than those in Ghana (45.1%) and Nigeria (50.8%) [25]. This difference could stem from variations in study periods, sample sizes, design, and social disparities between countries. The overall low compliance with IPC practices highlights the urgent need for policymakers, the health management team, and healthcare workers to strengthen strategies that promote the implementation of IPC practices and increase overall compliance.

More than half of HCWs in the current study reported inconsistent use of PPEs, such as surgical masks, goggles, face shields, and aprons, even when there was a risk of splash exposure. Additionally, most HCWs relied on water alone for hand washing despite the availability of IPC resources, including alcohol-based hand rub, soap, and detergents. Similar findings have been reported in Ethiopia, Uganda, and India, where poor PPE use and inadequate hand hygiene were linked to inconsistent PPE supply, discomfort, and perceived low risk [27–29]. The observed finding in this study reflects the ongoing inconsistent PPE supply and time constraints (high patient workloads, limited staffing) prevailing at tertiary hospitals in LMICs that likely contribute to HAI and AMR. In contrast, the Kenyan study found that HCWs followed IPC practices due to ongoing IPC training and strict supervisory checks, particularly during the COVID-19 era [30]. Studies previously demonstrated that access to hand hygiene items, along with multimodal promotion such as training and education of HCWs and monitoring and feedback on HH practices, was critical to improving hand hygiene and overall compliance, in both developed and developing countries’ healthcare settings [31].

In this study, doctors and clinicians showed notably lower compliance with IPC compared to nurses. Similar results have been reported by other studies in the United Kingdom, Pakistan, Bangladesh, Australia, and Songwe, Tanzania [26,32–34]. Plausible explanations for the current study may be associated with multiple factors. For nurses, for example, the inclusion of additional IPC courses in their training programs may contribute by equipping them with basic knowledge before they are enrolled in service. Also, it is speculated that nurses are more directly involved in patient care, and therefore, IPC is more relevant [35]. The observed finding may indicate the urgent need for targeted IPC educational training among healthcare workers, irrespective of cadres. Training should also be integrated into existing workflows to bridge the compliance gap and improve overall infection control outcomes at KRRH and similar healthcare settings. However, the shortage of doctors in many health facilities, including the study setting, is another possible explanation for low IPC compliance among doctors, resulting in an excessive workload and a corresponding decline in doctors’ abilities to follow IPC guidelines. These findings align with other studies that have found staffing ratios to negatively impact IPC. The findings signify that some of the major barriers to IPC compliance may depend on administrative changes, such as hiring additional staff to reduce workload. Strategies are needed to accommodate patient surges to ensure IPC can be maintained during and even after an epidemic crisis, where HCWs can otherwise become a hot spot for disease spread.

Healthcare workers who were exposed to body fluids were 1.5 times more likely to comply with IPC practices than those who were not exposed, implying that heightened risk perception is a strong driver of compliance. These findings are in line with other studies conducted in Ethiopia, which showed that HCWs with higher perceived risk had markedly better compliance with IPC guidelines [36]. This significant observation in this study underscores the importance of strengthening risk perception through continuous education and awareness, and acts as a call to action for hospital management and HCWs to reinforce compliance with IPC practices even in the absence of immediate exposure risks. In contrast to this observation is the study conducted in Ghana, which showed that HCWs who encountered blood exposure had lower compliance with the IPC guidelines [37]. The difference in findings may be due to differences in institutional policies and the coexistence of a weak safety culture in most resource-limited facilities. However, exposure alone is insufficient without system support [38].

Although the current study showed no significant association between IPC compliance and IPC training, training is important in enhancing knowledge of IPC protocols and practices [3]. More than 60% of HCWs reported not receiving any form of IPC training despite the country experiencing different seasons of emerging and re-emerging infectious diseases. A study from Bangladesh also highlighted that 85% of HCWs received no formal training on infection control [39]. Regular IPC training was also found to be lacking in studies in Ethiopia, Ghana, Pakistan, Australia, and even parts of Tanzania [25,33,40,41]. These findings demonstrate that the importance of regular IPC training may not be well understood by the hospital administration, or that IPC experts need to facilitate such training, which ultimately led to low compliance with IPC practices. The hospital should regularly arrange motivation sessions and hands-on IPC training for all staff to sustain and improve IPC compliance.

Healthcare workers who received IPC motivation at the workplace in the current study were more likely to comply with IPC practices than those not motivated. These findings are in line with a study conducted by Houghton et al, who reported that self-motivation among HCWs by perceiving IPC value of protecting themselves, their families, or their patients is a behavioural change motive towards IPC compliance [42]. Moreover, a supportive workplace culture and managerial backing further reinforce compliance among motivated staff [43]. Thus, intervention involving incentives, non-monetary, and recognition should be encouraged for improving IPC compliance among HCWs at KRRH and similar settings.

Furthermore, monitoring of the IPC practices via supportive supervision plays a vital role in enhancing compliance with the IPC practices. This observation forms the basis for the current study. Our study revealed that HCWs who received IPC supportive supervision were more likely to comply with IPC practices compared to those with no supportive supervision. These findings align with studies done in Gambia [42,44]. During supportive supervision, IPC activities are monitored, implementation gaps are identified, and addressed. Therefore, the findings highlight a need for a continuous and thorough monitoring system for IPC practices in tertiary-level healthcare facilities in Tanzania. This approach should be carried out regularly and in a non-punitive way, focusing not only on helping identify compliance gaps but also on motivating HCWs to consistently follow IPC practices.

Additionally, the current study revealed that HCWs who confirmed the presence of the IPC committee were more likely to comply with IPC practices compared to those who reported the absence of the IPC committee. A similar study done across health facilities in the Kampala region during the COVID-19 era emphasized that for the HCWs to comply with IPC practices, there is a need for IPC committee activities and work plans to be supported by the budget lines [45]. This observation presses the need for strengthening the IPC committee to actively perform their tasks, such as routine monitoring, providing feedback on IPC strategies, and HCWs sensitization on IPC practices, which may greatly contribute to overall IPC compliance.

### Strengths and limitations of the study

The strength of this study lies in its approach of using multiple data collection tools: questionnaires and observational checklists to gather data directly from the required participants, giving valuable insights for healthcare workers, health administrators, and policymakers. Additionally, the study identified several key findings that need further research with a larger sample and a more robust design, such as a longitudinal study, direct observation, and qualitative perspectives.

Despite the strength, this study has certain limitations. Initially, being a cross-sectional study makes it difficult to infer causality. Secondly, the measurement of IPC compliance relied on self-reported responses from the participants, which may introduce the possibility of social desirability bias influencing the accuracy of the data. Furthermore, only participants directly involved in patient care were included in the study, thus limiting generalizability. Although these limitations may have introduced some bias or reduced the study’s generalizability, their overall impact on the study’s conclusions is considered minimal.

## Conclusion and recommendation

Overall compliance with IPC practices among HCWs at Katavi Regional Referral Hospital was suboptimal relative to recommended standards for safe patient care. The findings suggest that factors such as supportive supervision, workplace motivation, and the presence of a functional IPC committee are associated with improved compliance. Identified gaps in WASH infrastructures, particularly inconsistent availability of water and soap in latrines, may further constrain compliance with IPC practices. Given a high burden of HAI and AMR, further studies should be carried out in a longitudinal study design involving multiple healthcare settings to validate these associations and better inform national-level IPC policy and implementation strategies.

## Supporting information

S1 Table Compliance with IPC Standard Precautions. (XLSX)

S1 Data IPC quantitative data set. (RAR)

## Abbrevations

AMR: Antimicrobial Resistance
HAIs: Hospital-Acquired Infections
HBV: Hepatitis B Virus
HH: Hand Hygiene
IPC: Infection Prevention and Control
KRRH: Katavi Referral Regional Hospital
PPEs: Personal Protective Equipment
